# Designed Azolopyridinium Salts Block Protective Antigen Pores *In Vitro* and Protect Cells from Anthrax Toxin

**DOI:** 10.1371/journal.pone.0066099

**Published:** 2013-06-20

**Authors:** Christoph Beitzinger, Anika Bronnhuber, Kerstin Duscha, Zsuzsanna Riedl, Markus Huber-Lang, Roland Benz, György Hajós, Holger Barth

**Affiliations:** 1 Rudolf-Virchow-Center, DFG-Research Center for Experimental Biomedicine, University of Würzburg, Würzburg, Germany; 2 Institute of Pharmacology and Toxicology, University of Ulm Medical Center, Ulm, Germany; 3 Institute of Organic Chemistry, Research Centre for Natural Sciences, Hungarian Academy of Sciences, Budapest, Hungary; 4 Institute of Traumatology, Hand- and Reconstructive Surgery, University of Ulm Medical Center, Ulm, Germany; 5 School of Engineering and Science, Jacobs University Bremen, Bremen, Germany; Institut Curie, France

## Abstract

**Background:**

Several intracellular acting bacterial protein toxins of the AB-type, which are known to enter cells by endocytosis, are shown to produce channels. This holds true for protective antigen (PA), the binding component of the tripartite anthrax-toxin of *Bacillus anthracis*. Evidence has been presented that translocation of the enzymatic components of anthrax-toxin across the endosomal membrane of target cells and channel formation by the heptameric/octameric PA_63_ binding/translocation component are related phenomena. Chloroquine and some 4-aminoquinolones, known as potent drugs against *Plasmodium falciparium* infection of humans, block efficiently the PA_63_-channel in a dose dependent way.

**Methodology/Principal Findings:**

Here we demonstrate that related positively charged heterocyclic azolopyridinium salts block the PA_63_-channel in the µM range, when both, inhibitor and PA_63_ are added to the same side of the membrane, the cis-side, which corresponds to the lumen of acidified endosomal vesicles of target cells. Noise-analysis allowed the study of the kinetics of the plug formation by the heterocycles. *In vivo* experiments using J774A.1 macrophages demonstrated that the inhibitors of PA_63_-channel function also efficiently block intoxication of the cells by the combination lethal factor and PA_63_ in the same concentration range as they block the channels *in vitro*.

**Conclusions/Significance:**

These results strongly argue in favor of a transport of lethal factor through the PA_63_-channel and suggest that the heterocycles used in this study could represent attractive candidates for development of novel therapeutic strategies against anthrax.

## Introduction

The main virulence factors of *Bacillus anthracis* are the poly-D-glutamic acid capsule, which inhibits phagocytosis, and anthrax toxin. The plasmid-encoded tripartite anthrax toxin comprises a receptor-binding and transport component termed protective antigen (PA) and two enzymatically active components termed edema factor (EF) and lethal factor (LF) [1]–[3]. PA binds to cells, coordinates self-assembly of heptamers and/or octamers on the cell surface, triggers endocytosis of the toxin complexes and finally delivers EF and LF from endosomal vesicles to the cytosol of the target cell [4]–[10]. This translocation scheme is common to many so-called binary AB-toxins including anthrax-, C2 and iota toxin [11]. EF is a calcium and calmodulin-dependent adenylate-cyclase (89 kDa) that causes a dramatic increase of intracellular cAMP level, upsetting water homeostasis and destroying the balance of intracellular signaling pathways [12], [13]. In addition, EF is believed to be responsible for the edema found in cutaneous anthrax [2], [14], [15]. LF is a highly specific zinc metalloprotease (90 kDa) that removes specifically the N-terminal tail of mitogen-activated protein kinase kinases (MAPKKs) [16]–[18]. This cleavage initiates still poorly understood mechanisms leading to subsequent cell death by apoptosis. The correlation between MAPKK cleavage and the LF dependent inhibition of the release of pro-inflammatory mediators like nitric oxide, tumor necrosis factor-alpha and interleukin-1ß is an actual subject of particular interest [19]–[21].

PA is a cysteine-free 83**kDA protein that binds to one of the two anthrax toxin receptors on target cells which are TEM8 (tumor endothelial marker 8, *alias* ANTXR1) and CMG2 (capillary morphogenesis 2, *alias* ANTXR2) [22], [23]. Receptor-bound PA is processed by a furin-like protease to a 63**kDa fragment PA_63_ and a 20**kDa fragment, PA_20_. PA_20_ dissociates from the receptor bound PA_63_ and is released into the extracellular milieu. PA_63_ then spontaneously oligomerizes into a heptamer or octamer [4], [8] and binds up to three molecules of EF and/or LF with high affinity (K_d_ ∼1**nM) [24]–[28]. The assembled toxic complex is then internalized by receptor-mediated endocytosis and directed to acidified endosomes. There, low pH results in conversion of PA_63_ into its pore formation and insertion into endosomal membranes forming the trans-membrane PA_63_-channel. Finally, EF and LF translocate across the endosomal membrane from the endosomal lumen into the cytosol. The PA_63_-channels have extensively been studied within the last 20 years [23], [29]–[32]. It exists increasing evidence that the pore lumen of trans-membrane channels is the translocation pathway for the enzymatic components [23], [32]–[34]. The related binary C2 toxin from *Clostridium botulinum* and iota toxin from *Clostridium perfringens* share a comparable translocation mechanism [35], [36].

If the theory that the pore lumen of the trans-membrane channels is the translocation pathway for the enzyme components is correct then the block of these channels should also block intoxication of cells. In fact, *in vivo* and cell-based experiments with C2- and anthrax-toxins have shown that block of the channels by chloroquine and other positively charged heterocyclic molecules such as quinacrine and fluphenazine blocked intoxication [31], [37]–[39]. Similarly, other positively charged molecules such as derivatives of ß-cyclodextrin were also able to specifically block channel formation *in vitro* and intoxication by C2- and anthrax-toxins in living cells [40]. When the ß-cyclodextrin-derived blockers contain additional hydrophobic aromatic groups on the thio-alkyl linkers of positively charged amino group the blockers were even more effective in blocking of C2- and anthrax-toxins and even Iota toxin by increasing the mean residence time of binding to the channels formed by the binding components [41].

**Figure 1:**
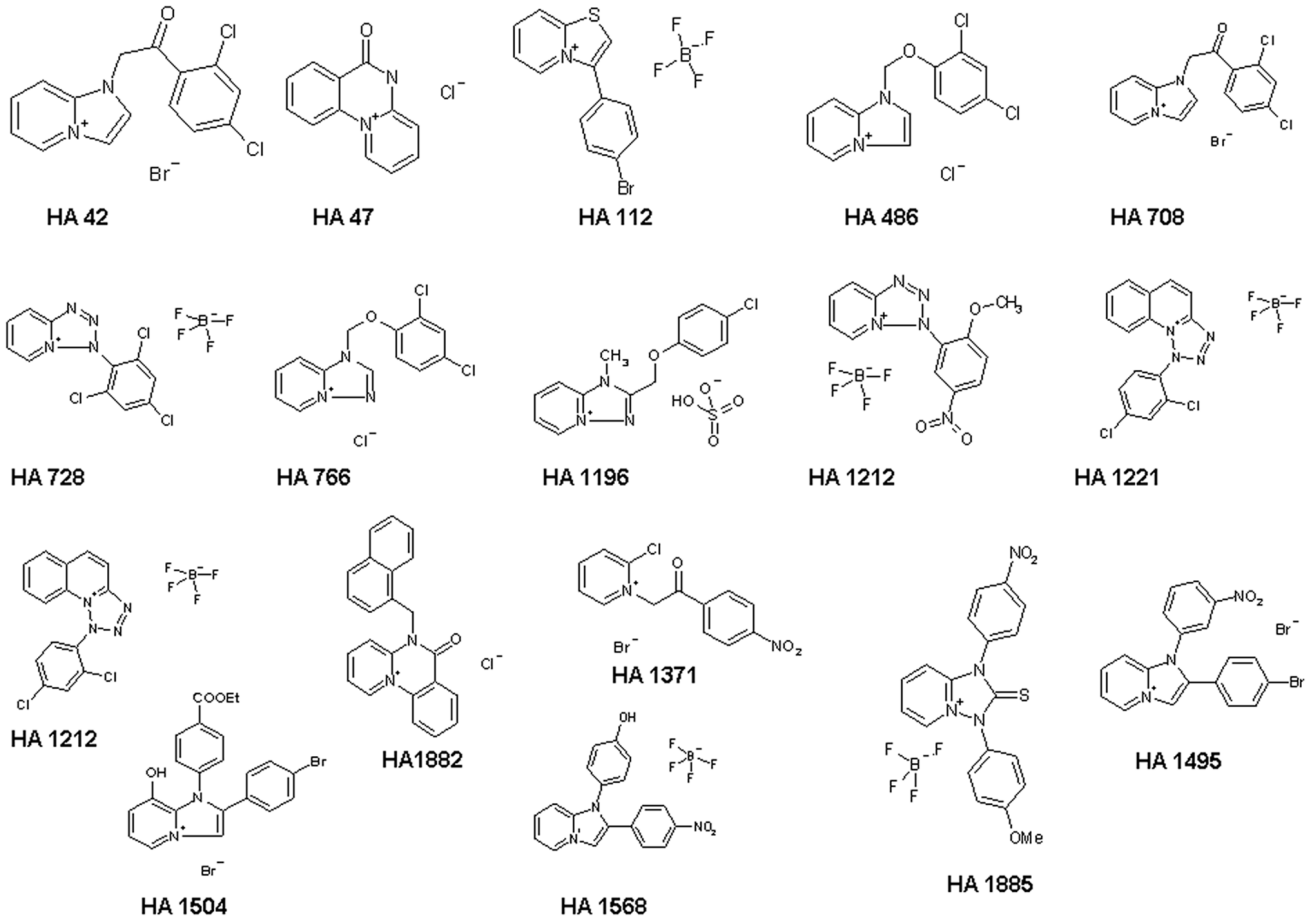
Structure of the heterocyclic chloroquine-related compounds used in this study. The counterions to the heterocycles are indicated. These derivatives have been synthesized according to methods described in the literature [62], [66]–[69].

In this study we investigated the binding properties of chloroquine-related heterocyclic fused azinium salts (see Fig. 1) to PA_63_-channels reconstituted in artificial membranes. Again the block of the PA_63_-channels resulted in a dose-dependent decrease of membrane conductance in titration experiments. The titration experiments provided interesting insight in the molecular requirement of azolopyridinium salts binding to the PA_63_-channel *in vitro*, which allowed the evaluation of the binding constants in fluctuation experiments based on a structure-function relationship of the used blockers [31]. On- and off-rate constants of their *in vitro*-binding to the PA_63_-channels were determined by the current noise analysis indicating a strong relationship between compound structures and binding kinetics to the PA_63_-channels. Cell-based experiments using J774A.1 macrophages suggested that the azolopyridinium salts also inhibit the intoxication of living cells by LF in combination with PA_63_ at µM concentration because they prevent the pH-dependent toxin translocation across cell membranes. Moreover, we demonstrate that the azolopyridinium salts have only negligible cytotoxic effects, which may allow their application *in vivo* to prevent intoxication by anthrax toxins.

## Results

### Binding of Chloroquinerelated Heterocyclic Azolopyridinium Salts to the PA_63_-channel

**Figure 2:**
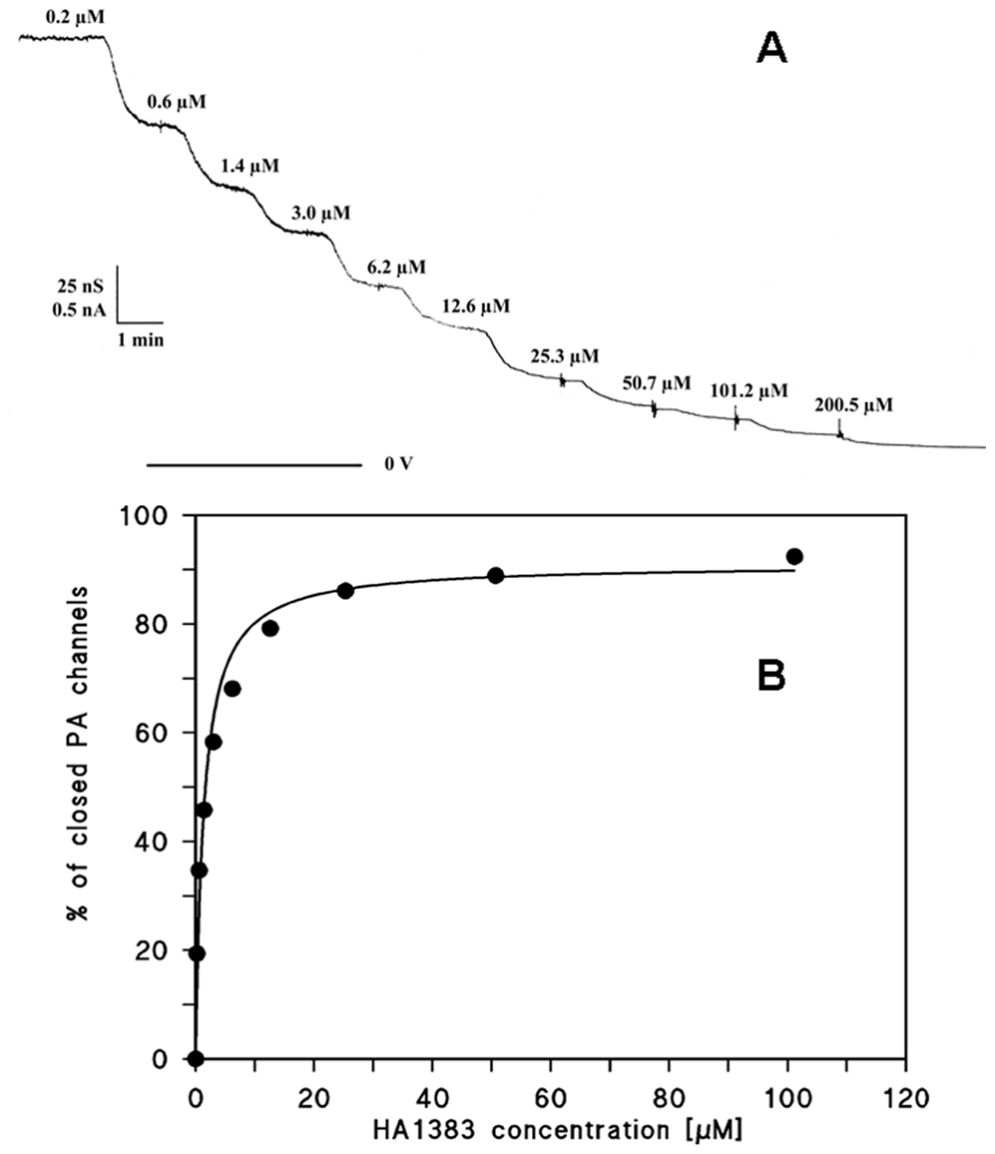
*A.* Titration experiment of PA_63_ induced membrane conductance with HA1383. The membrane was formed from diphytanoyl phosphatidylcholine/n-decane. The aqueous phase contained 1 ng/ml PA_63_ protein (added to the *cis*-side of the membrane), 150 mM KCl, 10 mM MES, pH 6.0 and HA 1383 in the concentrations shown on the top of the Figure. The temperature was constant at 20°C and the applied voltage was 20 mV. The membrane contained about 3,000 PA_63_-channels. The bottom line represents zero level of conductance. *B.* Lineweaver-Burke plot of the inhibition of PA_63_ induced membrane conductance by chloroquine and related blocker-substrates. The straight line corresponds to the data points taken from titration experiments in Fig. 2A. K = 740,000; 1/M A = 91%.

**Figure 3:**
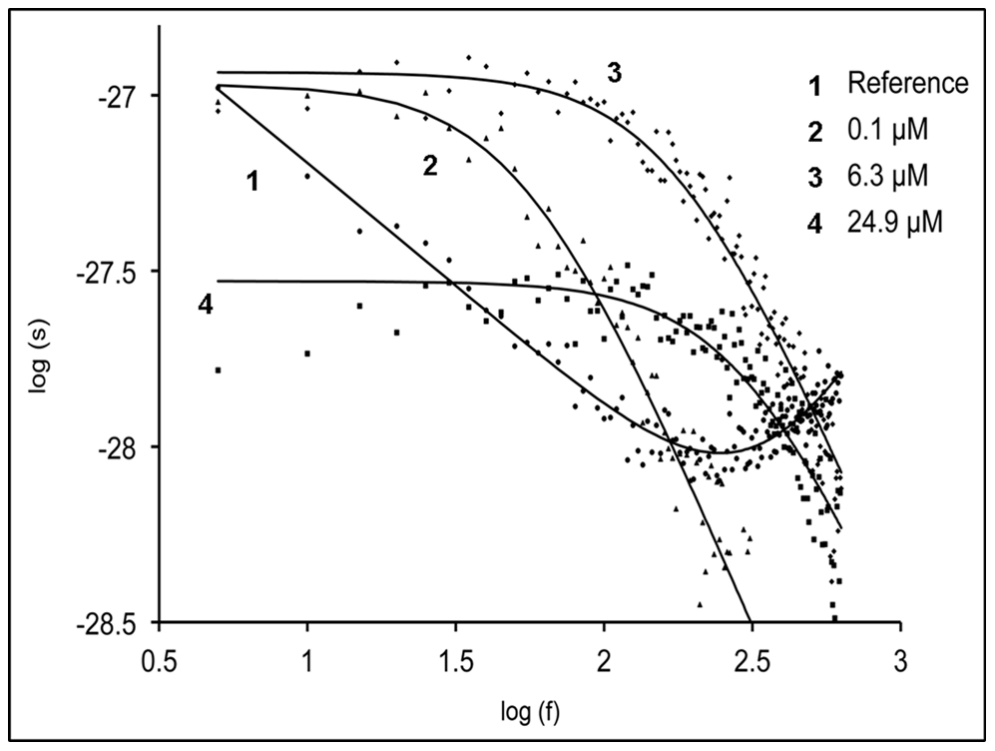
Power density spectra of HA1383 induced current noise of ∼450 PA_63_-channels (added to the *cis*-side). Trace 1 shows the control (PA_63_ without substrate). Trace 2–4: The aqueous phase contained 0.1, 6.3 and 24.9 µM HA1383, respectively and the power density spectrum of trace 1 was subtracted. Membrane was formed from diphytanoyl phosphatidylcholine/n-decane. The aqueous phase contained 150 mM KCl, 10 mM MES, pH 6.0, and about 1 ng/ml PA_63_ on the cis-side; V_m_ = 20 mV; T = 20°C.

The PA_63_-channel is fully oriented in artificial membranes when it is added to only one side of the membrane [29], [31]. In previous studies we demonstrated that reconstituted PA_63_-channels as well as C2II-channels can be blocked in lipid bilayer membranes by the addition of 4-aminoquinolines [31], [37], [39], [42]. The binding affinity strongly depends on negatively charged amino acids near the vestibule of PA_63_-channels. The stability constants *K* for substrate binding to the PA_63_-channels were calculated from multi-channel titration experiments. Activated PA_63_ was added to the *cis*-side (the side of the applied potential) of an artificial bilayer membrane while stirring. This led to a strong increase of membrane conductance caused by channel insertion in the membrane. After about 1 to 3 h, when the conductance was virtually stationary the titration of membrane conductance with the different heterocyclic azolopyridinium salts (HA-substances) was started. Small amounts of concentrated solution of one compound were added to the aqueous phase on both sides of the membrane while stirring to allow equilibration. Subsequently, the PA_63_-channels were blocked and a dose-dependent decrease of conductance was measured as a function of time. Fig. 2 shows an example for this type of measurements. After incorporation of 2.500 heptameric/octameric PA_63_-channels in a lipid bilayer membrane increasing concentration of substance HA1383 was added to both sides of the membrane to the aqueous phase (150 mM KCl, 10 mM MES, pH 6.0) while stirring to allow equilibration. The membrane current decreased in a dose-dependent manner. The titration curve given in Fig. 2 could be analyzed using a Lineweaver-Burke plot [31] or a Langmuir adsorption isotherm (see Fig. 3) [43]. Both analyses yielded in a stability constant *K* of 740,000 M^−1^ (half saturation constant *K_s_* of 1.34 µM) for the binding of HA1383 to the PA_63_-channels. The percentage of conductance that responded to ligand binding was 92% in the case of the experiment of Figure 2.

HA1363 had the lowest measured half saturation concentration of all heterocyclic compounds studied here, which is comparable to that of chloroquine *(K_s = _*1.43 µM) under the same conditions [31]. The half saturation constants of the other heterocyclic azolopyridinium salts were higher as compared to that of chloroquine. For HA1568, which possesses a high homology to HA1383 we observed almost the same half saturation concentration (*K_s_* = 2.7 µM). Other heterocyclic substances such as HA1196 and HA1371 revealed considerably higher half saturation constants (*K*_s_ = 260 µM and *K*_s = _310 µM respectively). In total we tested 17 heterocyclic azinium salts. The results are summarized in Table 1. The half saturation constants of the different heterocyclic derivatives range from *K_s_* = 1.3 µM for HA1383 to *K_s_* = 1.99 mM for HA47.

**Table 1:** Stability constants *K* and half saturation constants *K*_s_ for chloroquine and related blocker-substrates to PA-channels, when added to the *cis*-side of lipid bilayer membranes.

Heterocyclic compound	K [L/mol]	K_S_ [mmol/L]
HA 42	1460	0.69
HA 47	500	1.99
HA 112	18,500	0.057
HA 486	38,000	0.027
HA 708	11,300	0.089
HA 728	11,800	0.085
HA 766	29,400	0.036
HA 1196	3,800	0.26
HA 1212	7,000	0.15
HA 1221	6,000	0.17
HA 1371	3,200	0.31
HA 1383	749,000	0.0013
HA 1495	274,000	0.0036
HA 1504	82,900	0.012
HA 1568	478,000	0.0027
HA 1882	7,900	0.13
HA 1885	10,800	0.11
Chloroquine	676,000	0.0015

The data represent means of at least three individual titration experiments. *K*_s_ values from chloroquine are given for comparison and were taken from [31]. Membranes were formed from diphytanoyl phosphatidylcholine/*n*-decane. The aqueous phase contained 150 mM KCl, 10 mM MES, pH 6.0, and about 1 ng/ml PA_63_; T = 20°C.

### Analysis of the Heterocyclic Azolopyridinium Salts Induced Current Noise through the PA_63_-channel

**Figure 4:**
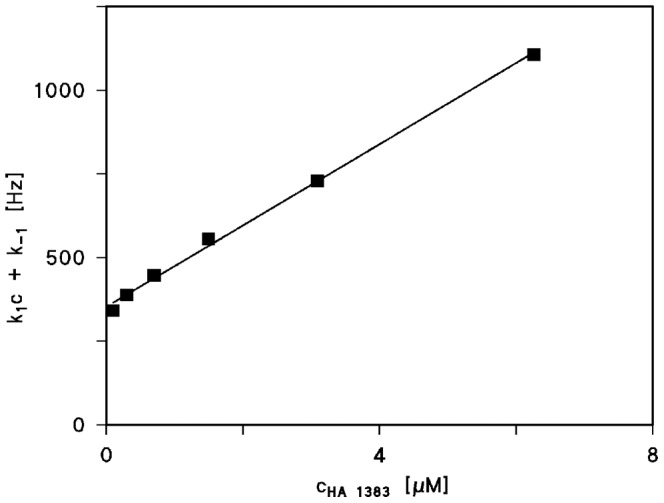
Plot of the corner frequencies of HA1383 induced current noise of PA_63_-channels as a function of the concentration of HA1383 on the both sides of the membranes. *k_1_* and *k_-1_* were derived from a fit of the corner frequencies as a function of the ligand concentration using eqn. (4).

The frequency-dependence of the spectral densities was measured using Fast-Fourier transformation of the current noise derived in parallel to some of the titration experiments. The measurement of the current noise required absolutely stationary conditions [44], [45]. Therefore, the time between reconstitution of PA_63_ and the start of the measurement was extended to about 2 h compared to standard titration experiments (about 30 min) to verify strictly stationary conditions. The reference spectrum was taken before addition of ligand to obtain the current noise spectrum of the open PA_63_-channels. The current noise spectra of open PA_63_-channels exhibited 1/ƒ noise in the frequency range between 1 and about 100 Hz (see Fig. 4; trace 1, Reference) similar to current noise of porins from enteric bacteria and open C2II-channels and previous noise measurements of open PA_63_-channels [31], [42], [44], [46]. For higher frequencies above about 200 Hz the spectral density of the current noise started to increase. This increase of the spectral density was caused by noise of the preamplifier and the membrane capacitance C_m_, which could easily be demonstrated by the measurement of the current noise of dummy circuits containing an appropriate capacitor. After recording of reference spectra we added HA1383 in increasing concentration to the aqueous phase with stirring to allow equilibration. The power density spectrum was measured again a few minutes after the current decayed to its new value and became stationary, which indicated complete equilibration of HA1383 within the aqueous phase. Trace 2 of Fig. 4 shows a power density spectrum taken at 0.1 µM HA1383. In further experiments the concentration of HA1383 was increased in defined steps. At another concentration of HA1383 (*c* = 6.3 µM) the power density spectrum corresponded to that of trace 3 in Fig. 4. Further increase of HA1383 to a concentration of 24.9 µM resulted in the power density spectrum of trace 4. The power density spectra of the current noise shown in Fig. 4 (Traces 2 to 4) corresponded to that of Lorentzian type and could be fitted to single Lorentzians after the subtraction of the reference spectrum (see Fig. 4). Such a type of noise is expected for a random switch with different on- and off-probabilities and can be fitted to equation (4) [31], [44], [45], [47], [48]:
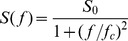
(4)


The corner frequencies, *f_c_*, of the Lorentzians are dependent on the on- and off-rate constant, *k_1_* and *k_-1_*, for ligand binding to the binding-site of the PA_63_-channel according to eqn. (5) [31], [44], [45]:
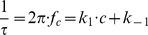
(5)


**Figure 5:**
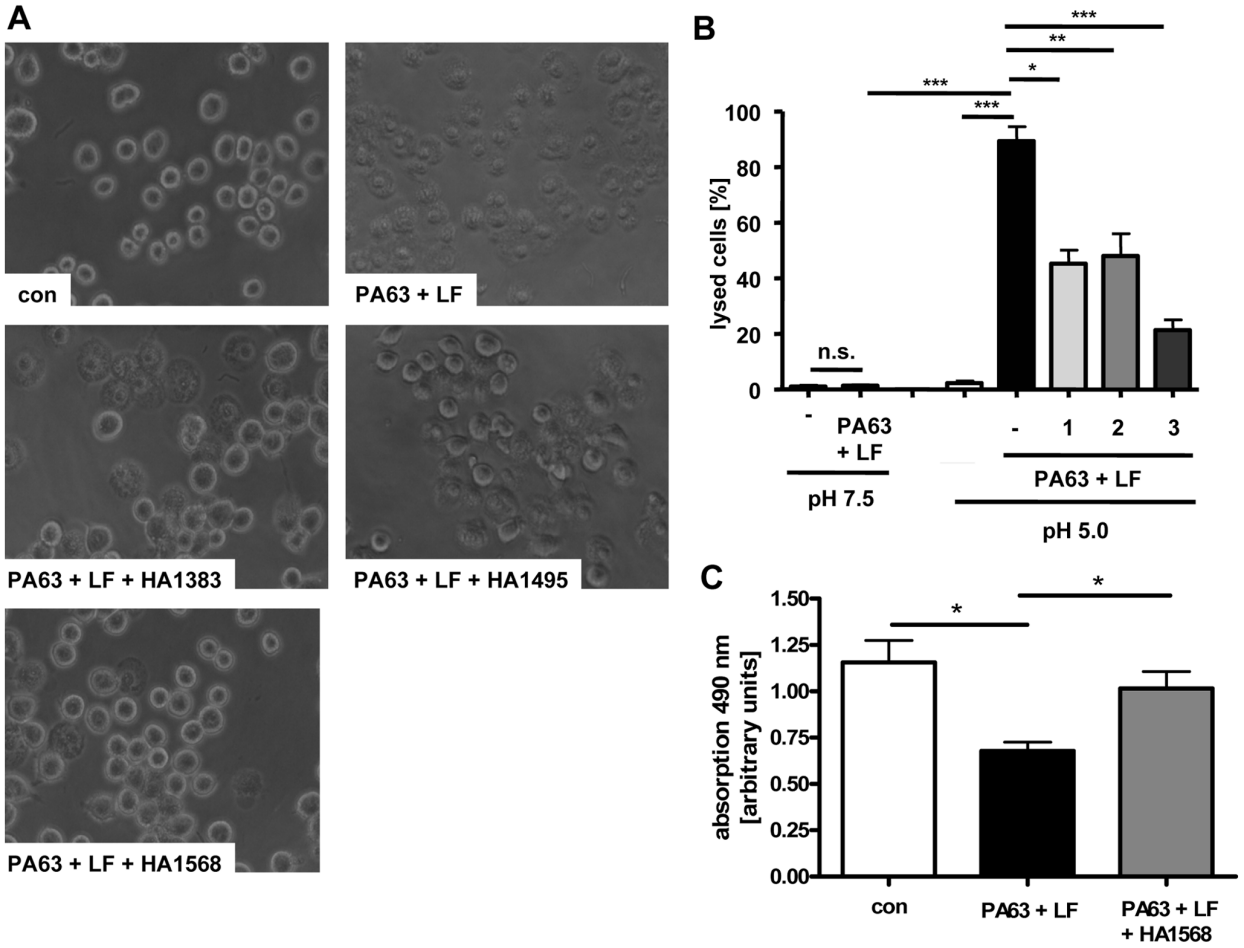
HA1383, HA1495 and HA1568 inhibit the PA_63_-mediated and pH-driven translocation of LF across the cytoplasmic membrane of J774A.1 macrophages. J774.A1 cells were incubated for 30 min at 37°C with bafilomycin A1 (100 nM) and some cells were also treated with 100 µM of either HA1383 (1), HA1495 (2) or HA1568 (3). Subsequently, the cells were incubated for 1 h at 4°C with PA_63_ (1 µg/mL) plus LF (1 µg/mL). Then, cells were incubated for 5 min at 37°C with acidic medium (pH 5.0) containing the respective HA substance. For control, cells were treated at pH 7.5 (not shown). Finally, cells were incubated at 37°C in neutral medium containing bafilomycin A1. After further 2 h of incubation pictures were taken. *A.* Pictures from cells that were treated with acidic medium. *B.* The percentage of lysed cells was determined from the pictures. Data are given as mean ± S.D (n = 3; n. s. = not significant; * = p<0.05; ** = p<0.005; *** = p<0.0005). *C.* After 2 h of incubation cell viability was determined. MTS/PMS solution (*Cell Titer 96® Aqueous non-radioactive cell proliferation assay* from Promega) was added into the medium at 1 h after the acidic shift and cells were incubated for an additional h at 37°C before the absorption was determined at 490 nm in an ELISA reader. Values are given as mean ± S. D. (n = 3; * = p<0.05).

This means that the corner frequencies, *f_c_*, should increase with increasing HA1383 concentration. This was the case for all noise measurements including the experiments shown in Fig. 4. The reaction rate *1/τ* was plotted as a function of the HA1383 concentration in the aqueous phase. Fig. 5 shows the fit of 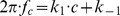 of the experiments shown in Fig. 4 and of other HA1383 concentrations (data not shown) to eqn. (5). The on-rate constant was calculated from a least-squares fit of the experimental data to be 1.2×10^8^ 1/(M·s), which is within the range of diffusion limited processes. The off-rate for HA1383 was very slow (*k_-1_* = 352 s^−1^), suggesting that HA1383 forms a stable plug in the PA_63_-channel. The corresponding stability and half saturation constant *K* and *K_s_*, respectively, are in good agreement with the binding parameters derived from titration experiments (see Table 2). Similar results were obtained from noise experiments using the heterocyclic azolopyridinium salt HA1568 with the second highest binding affinity to PA_63_. This compound had the strongest protective effect in cell-based experiments (see below). In this case the on rate constant *k_1_* was 1.3×10^8^ 1/(M·s) and the off-rate *k_-1_* 260 1/s, quite comparable to the binding kinetics of HA1383 (see Table 2).

**Table 2:** Parameters of HA1383-, HA1568- and HA486-induced current noise in PA_63_channels.

Heterocycle	*K* [L/mol]	*K_S_* [µmol/L]	*k_1_* [L/(molxs)]	*k_-1_* [1/s]	K* [L/mol]
HA1383	560,000	1.8	2.1×10^8^	370	749,000
HA1568	511,000	2.0	1.3×10^8^	260	478,000
HA486	34,000	32.0	1.2×10^8^	3,500	38,000
Chloroquine	1,030,000	0.97	3.6×10^8^	350	676,000

*k_1_* and *k_-1_* were derived from a fit of the corner frequencies as a function of the ligand concentration. *K* is the stability constant for ligand binding derived from the ratio *k_1_*/*k_-1_*. The data represent means of at least three individual experiments with the same substrate. Membranes were formed from diphytanoyl phosphatidylcholine/n-decane. The aqueous phase contained 150 mM KCl, 10 mM MES, pH 6.0, and about 1 ng/ml PA_63_ on the cis-side; T = 20°C. The data for chloroquine are given for comparison and were taken from [31].

Further experiments to study the current noise of blocking of the PA_63_-channel with other heterocyclic substances were only performed for HA486, which had a considerably lower binding affinity to PA_63_. These measurements were performed to understand the difference in binding kinetics to the highly effective blockers HA1383 and HA1568. Measurements with some other blockers were not successful because of the shift of the corner frequency of the Lorentzians to higher values, presumably because the off-rate constants *k_-1_* of complex formation between PA_63_-channel and the blockers were much higher than that for HA1383 and HA1568. In fact, the corner frequencies of the Lorentzians of current noise analysis for binding of HA486 to PA_63_ were in the range between about 800 to 3000 Hz. This means that the kinetics of HA486 binding to PA_63_ were considerably faster. On average we found for the on-rate of HA486 binding a value of *k_1_* = 1.2×10^8^ 1/(M·s), which is comparable to the on-rate of HA1383 and HA1568 binding. However, the off-rate *k_-1_* was with 3,500 1/s considerably higher than that for HA1383 or HA1568, which is clearly responsible for the different binding affinity of the two other ligands to the PA_63_-channels.

### Block of Anthrax Intoxication with Heterocyclic Azolopyridinium Salts in Cell-based Assays

The lipid bilayer experiments allowed a rapid analysis of the block of the PA_63_-channels by the chloroquine-based heterocyclic azolopyridinium salts. Prompted by the observation that HA1383, HA1495 and HA1568 most efficiently blocked PA_63_-channels *in vitro*, we finally tested whether these substances were able to block PA_63_-channelsin membranes of living cells and inhibited the pH-dependent membrane translocation of the enzyme component LF through these channels. To this end, we mimicked the toxin translocation which normally occurs from acidified endosomal vesicles to the cytosol on the surface of intact cultured J774A.1 macrophages as described earlier [49], [50]. The “normal” translocation of LF from endosomes is inhibited by bafilomycin A1 which prevents acidification of the endosomal lumen [51]. Cell-bound PA_63_/LF was exposed to an extracellular acidic pulse, which triggers insertion of PA_63_ into the cytoplasmic membrane where it forms trans-membrane channels. The PA_63_-bound LF then translocates through the PA_63_–channels across the cytoplasmic membrane into the cytosol where it induces lysis of J774A.1 macrophages [52], as shown in Fig. 5A. As shown in Fig. 5B, LF-induced cell lysis was observed only under acidic but not under neutral conditions. In the presence of each heterocyclic substance in the culture medium, the amount of lysed cells was significantly reduced (Fig. 5), implying that the heterocyclic substances inhibited membrane translocation of LF. Moreover, the protective effect of the heterocyclic substances against PA_63_/LF became also obvious when viability of J774A.1 cells was determined by the MTS-assay [53], as shown for the most effective blocker HA1568 in Fig. 5C.

**Figure 6:**
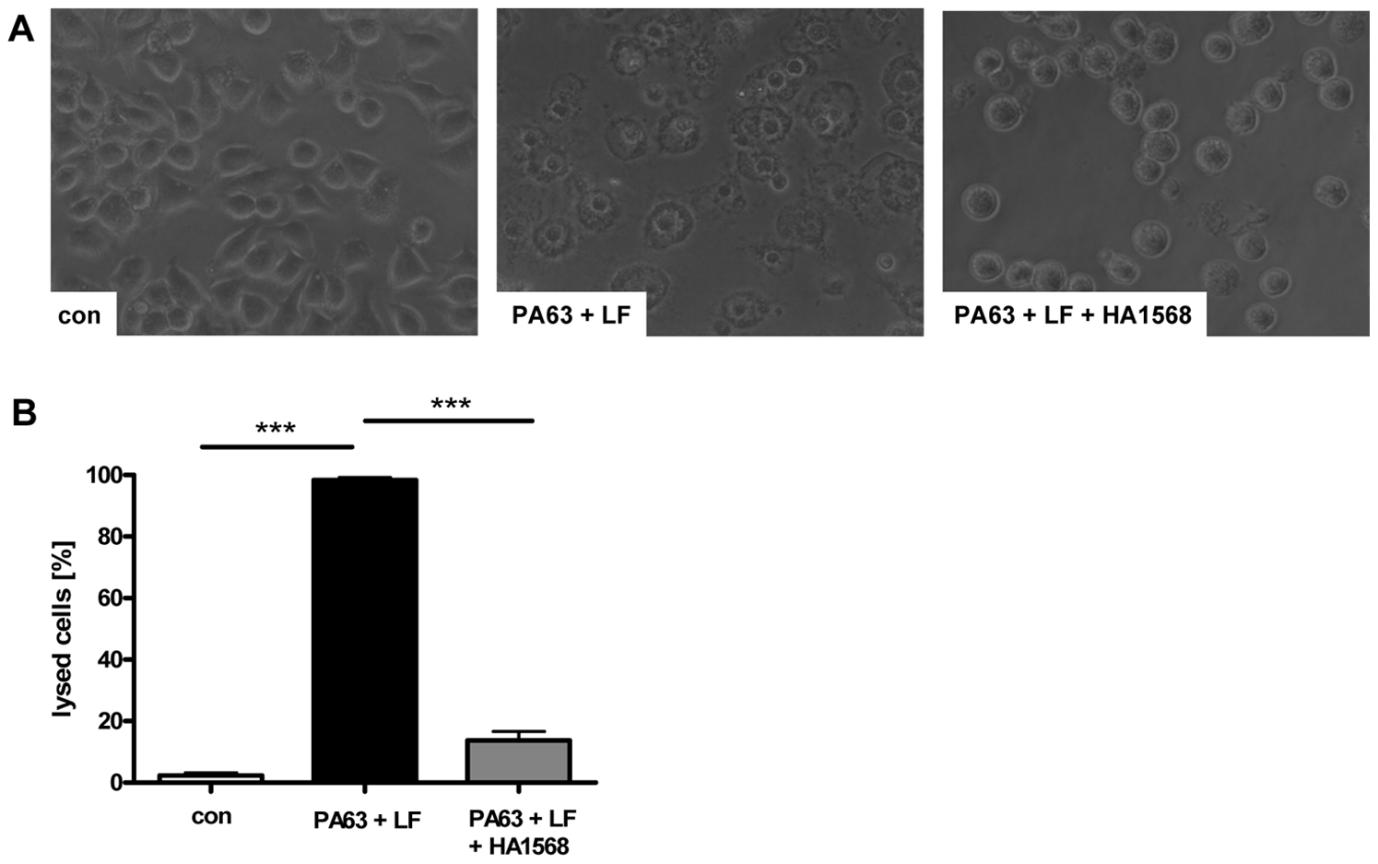
HA1568 protects J774A. 1 cells against the cytotoxic effect of PA63/LF. J774.A1 cells were incubated for 30 min at 37°C with 100 µM of HA1568 and subsequently PA_63_ (1 µg/mL) plus LF (1 µg/mL) were added to the medium. *A.* Cells were further incubated in the presence of the toxin and HA1568 at 37°C and pictures were taken after 2 h. *B.* The percentage of lysed cells was determined from the pictures. Data are given as mean ± S.D (n = 3; *** = p<0.0005).

**Figure 7:**
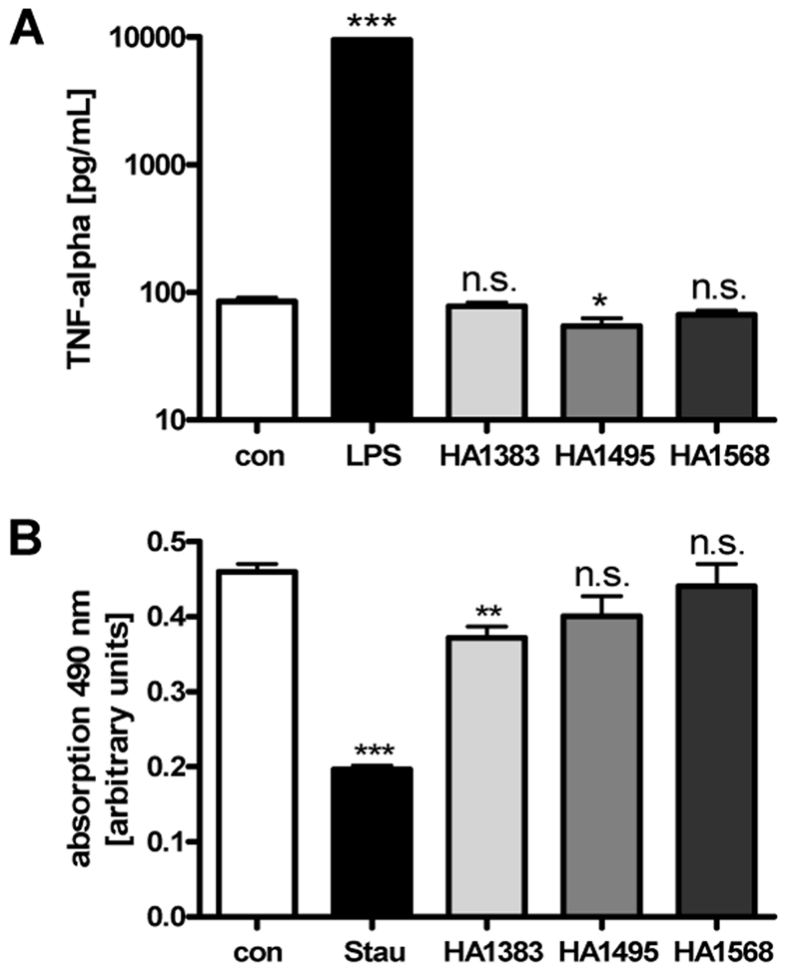
Effect of HA1383, HA1495 and HA1568 on viability of J774A.1 cells and TNFα-release. *A.* Cells were grown in 12-well plates and incubated for 24 h at 37°C with 100 µM of either HA-substance. For control, cells were left untreated. For positive control, cells were incubated with *E. coli* BL21 lysate.The concentration of TNFα in the medium was determined by ELISA assay. Values are given as mean ± S. D. and significance was tested for each sample against the untreated control. (n = 3; n.s. = not significant; * = p<0.05; *** = p<0.0005). *B.* Cells were grown in a 96-well plate and incubated for 24 h at 37°C with 100 µM of either HA-substance. For control, cells were left untreated. For positive control, cells were incubated with staurosporine. Cell viability was measured with the MTS cell proliferation assay. Values are given as mean ± S. D. and significance was tested for each sample against the untreated control. (n.s. = not significant; n = 3; ** = p<0.005; *** = p<0.0005).

In line with this result, the heterocyclic substances protected J774A.1 cells from lysis when the cells were incubated with PA_63_/LF in the medium at 37°C for 3 h. This is shown in Fig. 6 for HA1568 because this substance showed the strongest inhibitory effect in the acidic shift assay before. Although the inhibition of the cytotoxic mode of action of PA_63_/LF by HA1383 and HA1495 was less effective compared to HA1568, these compounds had a moderate inhibitory effect, too (not shown). Noteworthy, the HA-substances alone showed no effect on the morphology of J774A.1 cells (data not shown) and did not interfere with release of tumor necrosis factor alpha (TNFα) from these cells after a 24 h incubation period with 100 µM of the substances as determined by ELISA assay (Fig. 7A). This indicates that the HA-substances did not induce macrophage activation under these experimental conditions. A 24 h incubation of J774A.1 cells with 100 µM of HA1495 or HA1568 had no effect on cell viability as determined by MTS assay while treatment with 100 µM of HA1383 had no effect on cell viability after 7 h but slightly decreased it after 24 h (Fig. 7B).

Taken all together, azolopyridinium salts HA1383, HA1495 and HA1568 prevent intoxication of J774A.1 macrophages by anthrax lethal toxin (PA_63_+ LF), most likely by blocking the PA_63_-channels and thus inhibiting the pH-dependent translocation of LF through the channels across cellular membranes.

## Discussion

Substances which block PA_63_channels in lipid bilayer membranes *in vitro* and in membranes of living cells such as chloroquine derivatives [31], [40], [41], [54]–[57] represent potential candidates for development of novel therapeutic strategies against anthrax disease. In the present report, we screened a series of heterocyclic azolopyridinium salts and other related azines for their ability to block the trans-membrane channel formed by PA_63,_ the receptor-binding component of *B. anthracis*. The chloroquine-based heterocyclic 4-aminoquinolines were originally developed for the blockade of type I export in bacteria where transport trough the TolC channel is involved. The new heterocyclic substances show some important advantages for potential *in vivo* application such as very low toxic activity on cells after prolonged incubation periods and rapid equilibration in tissues. First, the inhibitory effects of a series of azolopyridinium salts was analyzed in planar lipid bilayer membranes *in vitro* and the most efficient substances HA1383, HA1495 and HA1568 were subsequently tested for their ability to interfere with PA_63_-channels in membranes of living cells.

In cells, PA_63_-channels are absolutely essential for the translocation of LF and EF from the lumen of acidified endosomal vesicles into the cytosol of target cells. In the cytosol these enzymes cause their adverse effects which are the molecular basis underlying anthrax disease in animals and humans. Consequently, the pharmacological inhibition of toxin translocation by blocking the translocation channels should protect cells from intoxication and thus might be a therapeutic strategy to prevent anthrax. We used J774A.1 macrophages for these experiments because they are established target cells for anthrax lethal toxin (PA_63_+ LF) and undergo cell death accompanied by a characteristic change in their morphology after relatively short incubation periods with this toxin. The cell-based pH-shift experiment revealed strong evidence that HA1383, HA1495 and HA1568 indeed block the PA_63_-channels in cell membranes and thereby inhibit translocation of LF. Moreover, the three heterocyclic substances exhibited significant protective effects against lethal toxin when the macrophages were incubated together with the toxin, implying that these heterocyclic substances block the PA_63_ channels in membranes of acidified endosomal vesicles during “normal” toxin uptake, too. However, HA1568 was most effective and about 80% of the lethal toxin-treated cells survived in the presence of this substance. Therefore, HA1568 would be the optimal candidate of this series of the selected heterocyclic substances for further *in vivo* studies to investigate whether such substances could protect animals from anthrax after challenge with toxin-producing *B. anthracis*.

Because of the widely comparable cellular uptake mechanism and the comparable structures and functions of the binding/translocation components between anthrax toxins and the binary actin ADP-ribosylating toxins from Clostridia, the new pore blockers identified in this study might also act on the trans-membrane channels of C2- and the iota-like toxins. However, there are reported differences between the channels of C2- and iota toxin regarding their affinity towards chloroquine-derivatives [37] due to different charges in the vestibules of the C2IIa-channels and the Ib-channels and therefore the azolopyridinium salts might exhibit different effects on these proteins.

### Stability Constants for Ligand Binding to the PA_63_-channel

In previous studies we could already demonstrate that chloroquine and homologues bind to the binding component PA of anthrax-toxin from *B. anthracis* and inhibit channel formation *in vitro*. Similarly, we could show that related molecules inhibited channel formation by C2II and intoxication by C2 toxin at µM concentration in living cells [37], [42]. Protective antigen (PA) of anthrax toxin shares significant sequence identity (33%) with C2II, suggesting that the two proteins have similar modes of action. PA_63_ has been crystallized in its monomeric and heptameric/octomeric prepore forms [4], [8]. These forms bind to the target cell membrane and insert 14- or 16-stranded ß-barrels into the membrane. The mushroom shaped channel-forming complex of PA_63_ is highly asymmetric, since most hydrophilic material is localized on one side of the membrane, the *cis* side of lipid bilayer membranes or the surface of the target cell, and only a very small part of the oligomer is localized in the target cell membrane [4], [8]. The structure is similar to that of α-hemolysin of *Staphylococcus aureus* where the heptamer forms also some sort of vestibule [58].

In this study we performed titration experiments with imidazopyridinium salts and some other related azines and found a high affinity of these compounds to PA_63_ (see Table 1). The affinity increased in the series HA47, HA42, HA1196 until HA1568 and HA1383, which both had the highest affinity to PA_63_ at 150 mM KCl. It has to be noted that the concentrations of the imidazopyridinium salts with the highest affinity used in our experiments are in a range that should allow therapeutical treatment to inhibit *in vivo* intoxication of cells by anthrax toxin. In fact, HA1383, HA1568 and HA1495 efficiently inhibited also anthrax lethal toxin (PA_63_+ LF) intoxication in cell-based assays with a low cytotoxicity for the cells. In addition, the structure of the imidazopyridinium salts is such that they may easily permeate through lipid bilayer part of biological membranes, which may allow easy equilibration in organisms. This represents also the unique advantage of imidazopyridinium salts over the ß-cyclodextrin-derived blockers containing additional hydrophobic aromatic groups on the thio-alkyl linkers of positively charged amino groups, which presumably do not easily equilibrate across biological membranes in mammalian organisms.

### On and Off Rate Constants for Binding to Three Imidazopyridinium salts to PA_63_

Based on the titration experiments it is clear that blockage of the channel by the different ligands occurs in an association-dissociation reaction. Like porins of gram negative bacteria [44], [46] or channel forming toxins [42], [31], the open PA_63_-channels exhibited *1/f* noise that is probably related to size and structure of the channels and may be caused by short flickers of the current through the channels [46], [59]. The addition of HA1383, HA1568 and HA486 (and presumably all the other imidazopyridinium salts) lead to a complete change of the current noise caused by the blocking reaction. As a consequence its spectral density *S*(*f*) as a function of the frequency *_f_* increased considerably and changed from a 1/*f* function to a so-called Lorentzian function. The analyses of the Lorentzians and the calculation of the corner frequency *f_C_* allowed the evaluation of the rate constants and the stability constants for binding of three imidazopyridinium salts to the PA_63_-channel according to eqs. (5). The principle is based on a simple chemical reaction for ligand binding to the PA_63_-channel. We did not observe any indication for the binding of more than one ligand molecule at the same time to the binding-site, i.e. the occurrence of two Lorentzians. We are therefore convinced that our simple model provides a good description of ligand binding to the PA_63_-channel and of the blockage of the ion movement by their binding.

Previous experiments with PA-channel blockers clarified that for an adequate binding the positively charge and the bulky ring system play an important role [31], [42]. The on-rate for the binding of the three ligands to PA_63_ was between 1.2⋅10^8^ 1/(M⋅s) and 1.8⋅10^8^ 1/(M⋅s) in 150 mM KCl, which means that it did not vary much with the structures of the different ligands. These results suggest that the high on-rate of the two ligands is already close to that of diffusion-controlled reaction processes [60]. In contrast to the on-rate the off-rate of the binding of HA1383, HA1568 and HA486 showed much higher variations and differed by more than a factor of ten. These data illustrates that for increasing size of the different ligands *k_-1_* decreased. A high off-rate was obtained with a compound that has a smaller number of aromatic rings. On the other hand it is also clear that the substituent at the aromatic rings have a major impact on the binding of the imidazopyridinium salts. Highest binding affinity was observed for salts, which have a nitroxyl group bound to an aromatic ring. This group leads to a charge delocalization in the molecules that favors binding.

The membrane spanning-domain of PA_63_ comprises the residues E302 to S325 with a conserved pattern of alternating hydrophobic and hydrophilic amino acids and a total amount of 3 negatively charged residues orientated towards the channel-lumen (E302, E308 and D315). For C2 toxin site-directed mutagenesis of negatively charged residues located in the membrane spanning region did not show any significant effect on 4-aminoquinolones binding to the C2II-channel [61]. To summarize we conclude, that, like for C2 toxin, the vestibule of the PA_63_ on the cis-side contains the negatively charged residues which are involved in binding of the imidazopyridinium salts.

## Materials and Methods

Recombinant, nicked PA_63_ as well as LF from *B. anthracis* were obtained from List Biological Laboratories Inc. (Campbell, CA, U.S.A). One mg of lyophilized PA_63_ protein was dissolved in 1 ml 5 mM HEPES, 50 mM NaCl, pH 7.5 complemented with 1.25% trehalose. Aliquots were stored at −20°C. Dulbeccòs modified Eagle medium (DMEM) and fetal calf serum were obtained from Invitrogen (Karlsruhe, Germany). Cell culture materials were obtained from TPP (Trasadingen, Switzerland). Bafilomycin A1 was obtained from Calbiochem (Bad Soden, Germany) and staurosporine from Sigma-Aldrich (München, Germany). The heterocyclic azolopyridinium salts HA1383, HA1495 and HA1568 were dissolved in dimethyl sulfoxide as 100 mM stock solutions.

### Synthesis and Structure of Heterocyclic Compounds

**Figure 8:**
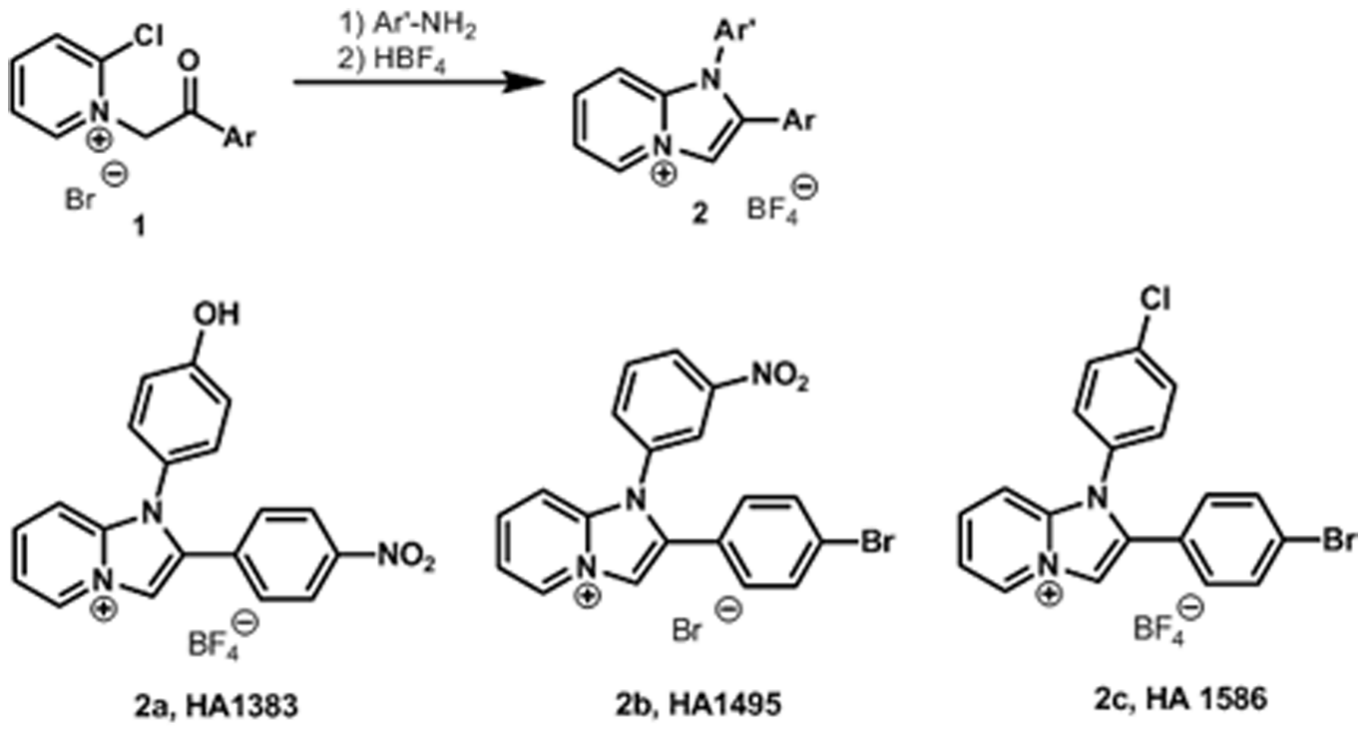
Synthesis and structure of the three heterocyclic model compounds HA1383, HA1495 and HA1568. 1-(4-hydroxyphenyl)-2-(4-nitrophenyl)imidazo[1,2-*a*]pyridinium tetrafluoroborate (HA1383); 1-(3-nitrophenyl)-2-(4-bromophenyl)imidazo[1,2-*a*]pyridinium bromide (HA1495); 1-(4-chlorophenyl)-2-(4-bromophenyl)imidazo[1,2-*a*]pyridinium tetrafluoroborate (HA1568).

Synthesis of the investigated imidazopyridinium salts (indicated as **2** in Fig. 8) has been carried out by ring closure of 2-chloro-N-phenacylpyridinium bromides with primary amines (Ar’-NH_2_) as shown in Fig. 8. The starting pyridinium salts (indicated as **1** in Fig. 8) were prepared by quaternization of 2-chloropyridine with substituted phenacyl bromides by application of a literature procedure [62]. As a result, three model compounds: 1-(4-hydroxyphenyl)-2-(4-nitrophenyl)imidazo[1,2-*a*]pyridinium tetrafluoroborate (**HA1383**), 1-(3-nitrophenyl)-2-(4-bromophenyl)imidazo[1,2-*a*]pyridinium bromide (**HA1495**), and 1-(4-chlorophenyl)-2-(4-bromophenyl)imidazo[1,2-*a*]pyridinium tetrafluoroborate (**HA1568**) have been isolated as shown in Fig. 8. The complete list of the fused azinium salts used in this study is shown in Fig. 1.

### General Procedure for Ring Closures to Imidazo[1,2-*a*]pyridinium Salts

To a solution of the appropriate 2-chloro-N-phenacyl bromide (1,1 mmol) in dimethyl formamide (10 ml) – this solution can be prepared at warm temperature but not exceeding 100 ^o^C - a solution of the selected amiline derivative (1.4 mmol) in dimethyl formamide (1 ml) was added, and the mixture was stirred at 100 ^o^C for 4 h. The mixture was then evaporated, water (2 ml) and hydrogen tetrafluoroborate (40%, 0.5 ml) was added and refluxed for 5 min. After cooling, the mixture was allowed to stand at room temperature; the separated solid was removed by suction filtration to yield the product.

### 1-(4-hydroxyphenyl)-2-(4-nitrophenyl)imidazo[1,2-*a*]pyridinium Tetrafluoroborate (2a, HA1383)

This compound was obtained from 2-chloro-1-(2-(4-nitrophenyl)-2-oxoethyl)pyridin-1-ium bromide (mp: 171-173 ^o^C) and 4-aminophenol to give white crystals (0.31 g, 75%, mp: 228-230 ^o^C). IR (KBr): 3437, 3119, 1643, 1599, 1519, 1345, 1278, 1220, 1104, 1048, 858 cm^−1^. ^1^H NMR (300 MHz, DMSO-d_6_) δ: 10.24 (s, 1H, H-OH), 9.06 (d, 1H, *J* = 6.6 Hz, H-5), 8.95 (s, 1H, H-3), 8.28 (m, 2H, H-3′-NO_2_-phenyl), 8.06 (t, 1H, *J* = 6.0 and 8,1 Hz, H-7), 7.70 (t, 1H, *J* = 6.6 and 8.1Hz, H-6), 7.69 (d, 1H, *J* = 6.0 Hz, H-8), 7.64 (m, 2H, H-2′-NO_2_-phenyl), 7.41 (m, 2H, H-2″-OH-phenyl), 6.96 (m, 2H, H-3″-OH-phenyl). δ_C_: 159.26, 148.21, 135.44, 135.19, 131.86, 130.65, 129.39, 124.00, 122.95, 118.46, 116.83, 111.55.

### 1-(3-nitrophenyl)-2-(4-bromophenyl)imidazo[1,2-*a*]pyridinium Bromide (2b, HA1495)

This compound was obtained from 1-(2-(4-bromophenyl)-2-oxoethyl)-2-chloropyridin-1-ium bromide (mp: 176-177 ^o^C) and 3-nitroaniline to give white crystals (0.32 g, 72%, mp:>300 ^o^C). IR (KBr): 3079, 1644, 1535, 1510, 1488, 1348 cm^−1^. ^1^H NMR (300 MHz, DMSO-d_6_) δ: 9.18 (d, 1H, *J* = 6.6 Hz, H-5), 8.99 (s, 1H, H-3), 8.70 (m, 1H, H-2″-*m*-NO_2_-phenyl), 8.50 (d, 1H, *J* = 6.0 Hz, H-8), 8.14 (t, 1H, *J* = 6.0 and 7.5 Hz, H-7), 8.00-7.86 (m, 3H, H-4″, H-5″ H-6″ *m*-NO_2_-phenyl), 7.72 (t, 1H, *J* = 6.6 and 7.5 Hz, H-6), 7.67 (m, 2H, H-2`-Br-phenyl), 7.33 (m, 2H, H-3`-Br-phenyl). δ_C_: 148.58, 140.81, 135.94, 135.54, 134.94, 133.20, 132.17, 131.83, 131.55, 125.64, 124.29, 124.11, 123.88, 118.71, 111.58.

### 1-(4-chlorophenyl)-2-(4-bromophenyl)imidazo[1,2-*a*]pyridinium Tetrafluoroborate (2c, HA1568)

This compound was obtained from 1-(2-(4-bromophenyl)-2-oxoethyl)-2-chloropyridin-1-ium bromide (mp: 176-177 ^o^C) and 4-chloroaniline to give white crystals (0.37 g, 78%, mp: 188-191 ^o^C). IR (KBr): 3094, 1645, 1530, 1514, 1494, 1063, 764 cm^−1^. ^1^H NMR (300 MHz, DMSO-d_6_) δ: 9.05 (d, 1H, *J* = 6.0 Hz, H-5), 8.83 (s, 1H, H-3), 8.05 (t, 1H, *J* = 7.5 Hz, H-7), 7.76-7.61 (m, 8H, H-6, 8 and H-*p*-Br-phenyl, H-3″-Cl-phenyl), 7.28 (m, 2H, H-2″-Cl-phenyl). δ_C_: 140.58, 135.97, 135.47, 135.35, 132.13, 131.35, 131.19, 130.47, 130.05, 129.55, 124.35, 124.26, 118.56, 111.44.

### Cell Culture, Cytoxicity Assay and Measurement of TNFα-release

DMEM culture medium and fetal calf serum were from Invitrogen (Karlsruhe, Germany) and HAM´s F12 from GIBCO (Karlsruhe, Germany). Cell culture materials were obtained from TPP (Trasadingen, Switzerland). J774A.1 macrophage-like cells (from DSMZ, Braunschweig, Germany) were cultivated at 37°C and 5% CO_2_ in DMEM containing 10% heat-inactivated fetal calf serum, L-glutamate (4 mM), penicillin (100 µg/mL) and streptomycin (100 µg/mL). The cells were cultivated at 37°C and 5% CO_2_. Cells were trypsinized and reseeded for at most 15-20 times. For cytotoxicity experiments, cells were seeded in 96-well-plates and incubated with PA_63_+ LF together with the respective heterocyclic azolopyridinium salt in serum-free medium. After the indicated incubation periods, the morphology of J774A.1 cells was visualized by using a Zeiss Axiovert 40CFl microscope (Oberkochen, Germany) with a Jenoptik progress C10 CCD camera (Jena, Germany). The cytotoxic effect caused by PA_63_/LF on this cell line was analyzed in terms of cell lysis. Cell viability of J774.A1 cells was determined by using the Cell Titer 96® Aqueous non-radioactive cell proliferation assay from Promega (Mannheim, Germany), according to the manufacturer’s instructions. For positive control, cells were treated with staurosporine, for negative control, cells were left untreated. To measure the release of TNFα after incubation with HA-substances, J774A.1 cells were incubated in 12-well plates for 24 h at 37°C with 100 µM of either substance in the medium. For negative control, cells were left untreated and for positive control, cells were incubated with *E. coli* BL21 lysate to induce TNFα-release. The medium (500 µL) was analyzed for TNFα-concentration by the enzyme linked immunosorbent assay (ELISA) from Becton, Dickinson and Company (Heidelberg, Germany), according to manufacturer’s instructions.

### Membrane Translocation Assay

The pH-dependent membrane translocation of LF through PA_63_-channels was investigated on the surface of intact J774.A1 cells as originally described earlier (1, 2). Cells were grown in 96-well-plates and incubated for 30 min at 37°C in serum-free medium with bafilomycin A1 (100 nM). During this period, some cells were treated also with 100 µM of either chloroquine-based heterocyclic azolopyridinium salts (HA substances). Subsequently, the cells were incubated for 30 min at 4°C with PA_63_ (1 µg/mL) plus LF (1 µg/mL) to enable binding of these proteins to the cell surface. Thereafter, the medium was removed and cells were incubated for 5 min at 37°C with acidic medium (pH 5.0, for control pH 7.5) containing the respective HA substance (100 µM). The acidic medium was removed and cells were incubated at 37°C in neutral medium containing bafilomycin A1 (100 nM) to prevent normal uptake of LF. Pictures were taken after 2 h and the toxin-induced cell lysis was analyzed exactly as described before.

### Lipid Bilayer Experiments

Black lipid bilayer experiments were performed as described previously [63] using a 1% solution of diphytanoyl phosphatidylcholine (Avanti Polar Lipids, Alabaster AL, U.S.A.) in n-decane as membrane forming lipid. The instrumentation consisted of a Teflon chamber with two aqueous compartments separated by a thin wall. The small circular whole between the two compartments had a surface area of about 0.4 mm^2^. The aqueous salt solutions were buffered with 10 mM MES, pH 6. All salts were obtained from Merck (Darmstadt, Germany). PA_63_ was added from concentrated stock solutions after the membrane had turned black, to the aqueous phase to one side (the *cis*-side) of the membrane. The PA-induced membrane conductance was measured after application of a fixed membrane potential with a pair of silver/silver chloride electrodes with salt bridges inserted into the aqueous phase on both sides of the membrane. The electrodes were connected in series to a voltage source and a home made current-to-voltage converter made with a Burr Brown operational amplifier. The amplified signal was monitored on a storage oscilloscope (Tektronix 7633) and recorded on a strip chart recorder (Rikandenki, Freiburg, Germany). The temperature was kept at 20°C throughout.

### Titration Experiments

The binding of heterocyclic azolopyridinium salts to PA_63_-channels was studied by titration experiments similar to those used previously to investigate binding of carbohydrates to the LamB-channel of *Escherichia coli* or binding of chloroquine or EF and LF, respectively, to C2II- and PA_63_-channels in single- or multi-channel experiments [27], [31], [33], [42], [64]. PA_63_-channels were reconstituted into lipid bilayer membranes from the *cis*-side of the artificial membrane and about 30–60 min after addition the rate of channel insertion decreased rapidly. Subsequently, concentrated solution of one heterocyclic azolopyridinium salts was added to both sides of the membrane while stirring to allow equilibration. Figure 2 shows an example of a titration experiment with heterocyclic azolopyridinium salt HA1383. The membrane conductance decreased as a function of concentration of the added substance. The data of the channel blockage were analysed similar as performed previously [64]. The conductance, *G(c)*, of a PA_63_-channel in the present of HA1383 or related substances with the stability constant, *K*, and the ligand concentration, *c*, is given by the maximum conductance (without ligand), *G*_max_, times the probability that the binding site is free.
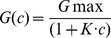
(1)


This equation may also be written as follows,
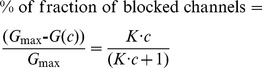
(2)


The half saturation constant *K_s_* is given by the inverse stability constant 1/*K*. The results of the titration experiments, i.e. the blockage of the channels as a function of the ligand concentration, were examined using the Langmuir adsorption isotherm [43], [64].

In most cases the different HA-substances caused full blockage the PA_63_-channels. In case of only partial blockage of the PA_63_-channels caused by a certain fraction of channels that did not respond to binding of the different substances (eqn. (2)) has to be modified to take the maximum degree of blockage into account (normally greater than 90%):
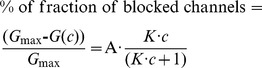
(3)


A is the maximum degree of blockage of the PA_63_-channel by the different ligands.

### Current-noise-analysis

The membrane current was measured with a current amplifier (Keithley 427 with a four pole filter or a home-made operational amplifier with a tree pole filter). Feedback resistors between 0.01 and 10 GΩ were used. The membrane current increased as a result of insertion of reconstituted PA_63_-channels. The amplified signal was simultaneously monitored by a strip chart recorder and fed through a low pass filter (4 Pole Butterworth Low-Pass Filter) into an AD-converting card of an IBM-compatible PC. The digitized data were analysed with a homemade fast Fourier transformation program, which yielded identical results as compared to a commercial digital signal analyser (Ono Sokki CF210, Addison, IL, U.S.A.). The spectra were composed of 400 points and averaged either 128 or 256 times. To analyse the data commercial graphic programs were used. For the derivation of the rate constants of ligand binding they were fitted to equations described in previously performed studies [44], [45], [64], [65].

### Reproducibility of the Experiments and Statistics

All experiments were performed independently at least 2 times. Results from representative experiments are shown in the figures.
